# Sleep Deprivation Deteriorates Heart Rate Variability and Photoplethysmography

**DOI:** 10.3389/fnins.2021.642548

**Published:** 2021-04-08

**Authors:** Nicolas Bourdillon, Fanny Jeanneret, Masih Nilchian, Patrick Albertoni, Pascal Ha, Grégoire P. Millet

**Affiliations:** ^1^Institute of Sport Sciences, University of Lausanne, Lausanne, Switzerland; ^2^be.care SA, Renens, Switzerland; ^3^MVT My-vitality sàrl, Founex, Switzerland

**Keywords:** heart rate variability, sleep, sleep deprivation, autonomic (vegetative) nervous system, photoplethysmography

## Abstract

**Introduction:**

Sleep deprivation has deleterious effects on cardiovascular health. Using wearable health trackers, non-invasive physiological signals, such as heart rate variability (HRV), photoplethysmography (PPG), and baroreflex sensitivity (BRS) can be analyzed for detection of the effects of partial sleep deprivation on cardiovascular responses.

**Methods:**

Fifteen participants underwent 1 week of baseline recording (BSL, usual day activity and sleep) followed by 3 days with 3 h of sleep per night (SDP), followed by 1 week of recovery with sleep *ad lib* (RCV). HRV was recorded using an orthostatic test every morning [root mean square of the successive differences (RMSSD), power in the low-frequency (LF) and high-frequency (HF) bands, and normalized power nLF and nHF were computed]; PPG and polysomnography (PSG) were recorded overnight. Continuous blood pressure and psychomotor vigilance task were also recorded. A questionnaire of subjective fatigue, sleepiness, and mood states was filled regularly.

**Results:**

RMSSD and HF decreased while nLF increased during SDP, indicating a decrease in parasympathetic activity and a potential increase in sympathetic activity. PPG parameters indicated a decrease in amplitude and duration of the waveforms of the systolic and diastolic periods, which is compatible with increases in sympathetic activity and vascular tone. PSG showed a rebound of sleep duration, efficiency, and deep sleep in RCV compared to BSL. BRS remained unchanged while vigilance decreased during SDP. Questionnaires showed an increased subjective fatigue and sleepiness during SDP.

**Conclusion:**

HRV and PPG are two markers easily measured with wearable devices and modified by partial sleep deprivation, contradictory to BRS. Both markers showed a decrease in parasympathetic activity, known as detrimental to cardiovascular health.

## Introduction

Reduction in sleep duration has deleterious effects on the neurocognitive functions (Drummond and Brown, 2001) and the cardiovascular system (Cappuccio et al., 2011). Partial sleep deprivation is associated with increased risk of hypertension (Gangwisch et al., 2006), infarctus, and stroke (Ayas et al., 2003), not to speak about the deficit in attention (Arnal et al., 2015), which may cause life-threatening accidents (Philip and Akerstedt, 2006). Therefore, detection of sleep deprivation is paramount for preventing diseases and accidents. It is also important that such detection be non-invasive, easy to use, and not time-consuming for the user.

Lack of sleep affects the sympathovagal balance, but results in the literature are discrepant regarding the nature of the changes. Sleep time reduction during five nights was sufficient to cause a significant increase in global sympathetic activity (Dettoni et al., 2012), while short sleep duration, low sleep efficiency, and insomnia were associated with lower levels of cardiac parasympathetic tone and higher levels of sympathetic tone (Zhong et al., 2005; Castro-Diehl et al., 2016; Jarrin et al., 2018). However, other contradictory studies have shown an increase in parasympathetic activity and a decrease in sympathetic activity (Holmes et al., 2002; Vaara et al., 2009) or no change (Glos et al., 2014) associated with sleep deprivation. Studies demonstrating a decreased sympathetic activity involved subjects in recumbent posture during the deprivation period or required subjects to minimize their physical activity (Mullington et al., 2009). Partial sleep deprivation is likely a lot more common in the general population than total sleep deprivation. Total sleep deprivation occurs in well-identified situations, such as night shift workers, whereas partial sleep deprivation may result from the very widespread sleep troubles, disturbed sleep due to ambient noise, light, increased screen time during the day, or lack of physical activities.

The easiest non-invasive way to assess the sympathovagal balance is to use heart rate variability (HRV) (Task Force, 1996). It allows to evaluate the sympathetic and parasympathetic modulations on the heart. The low-frequency (LF) band reflects a mix of sympathetic and parasympathetic influences (Holzman and Bridgett, 2017). Whether it may be considered an index of the sympathovagal balance (Goldberger, 1999) is a subject of debate (Eckberg, 1997). The LF power of HRV may be a measure of modulation of cardiac autonomic outflows by baroreflexes (Goldstein et al., 2011; Heathers, 2012). The migration of the respiratory sinus arrhythmia (RSA) from the high-frequency band (HF) into LF is a subject of debate (Wang et al., 2016), and it is one of the reasons that leave unclear the physiological implications of this frequency band (Medeiros et al., 2018; Hayano and Yuda, 2019). RSA is the main phenomenon inducing changes in the HF band and root mean square of the successive differences (RMSSD), which mainly reflects parasympathetic influences on the heart (Pomeranz et al., 1985). HRV-based methods are valid and reliable in the evaluation of stress and recovery (Teisala et al., 2014) and have successfully been associated with attention deficit during partial sleep deprivation (Henelius et al., 2014).

Complementary to HRV, baroreflex sensitivity (BRS) and photoplethysmography (PPG) are two techniques that have successfully assessed changes in the cardiovascular function associated with stress and fatigue (Bourdillon et al., 2019). A decrease in BRS is generally associated with deteriorated cardiovascular condition and high sympathetic tone while an increased BRS is associated with a healthy cardiovascular system and increased parasympathetic tone (Porta et al., 2018). So far, BRS has only been investigated with total sleep deprivation (Ogawa et al., 2003).

Modifications in the PPG waveform also reflect changes in the sympathovagal balance (Allen, 2007). For example, people with increased sympathetic activity and reported fatigue show decreased systolic amplitude (aS), diastolic amplitude (aD), and dicrotic amplitude (Bourdillon et al., 2019). However, to our knowledge, neither BRS nor PPG have been investigated during partial sleep deprivation.

Therefore, the aim of this study was to assess HRV, BRS, and PPG in healthy individuals undergoing three consecutive nights of partial sleep deprivation. It was hypothesized that HRV, BRS, and PPG would be modified by sleep deprivation; decreased parasympathetic modulations were expected, i.e., decreased HF, decreased BRS, and modified PPG waveform.

## Materials and Methods

### Design

The present study consisted in three consecutive phases: a baseline period of 1 week (BSL), a partial sleep deprivation period of 3 days (SDP), and a recovery period of 1 week (RCV). During BSL, the participants slept and lived as they used to in the preceding 3 months. During SDP, the participants slept 3 h per night on three consecutive nights, going to sleep late and waking up at their usual time. During RCV, the participants slept as much as they wanted. During the whole study, participants carried out their normal daytime activities.

**FIGURE 1:**
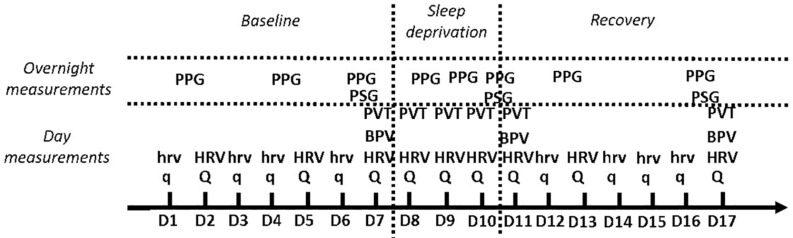
Illustration of the study protocol. q: Eight-item overtraining, Karolinska Sleepiness Scale Groningen Sleep Quality Scale questionnaires. Q: 14-item fatigue scale and Profile of Mood States questionnaires in addition to q. hrv: 3 min in supine position followed by 3 min in standing position orthostatic test. HRV: 6 min supine followed by 6 min standing orthostatic test. BPV, blood pressure variability; PVT, psychomotor vigilance task; PSG, polysomnography; PPG, photoplethysmography.

Figure 1 illustrates the protocol described hereafter.

### Participants

Fifteen participants performed the entire protocol in this study (eight women, seven men). The inclusion criteria were as follows: being in good general health with no sleep-related disturbance and not working with night shift duties or habits, no medication, and no pregnancy or lactation in the 3 months preceding inclusion in the study or during the study. The participants were young and physically active but not highly trained. All participants provided written informed consent prior to participation. The local ethics committee approved the study (agreement 2016-00308; Commission Cantonale d’Ethique de la Recherche sur l’être humain, CCER-VD, Lausanne, Switzerland). All experimental procedures conformed to the standards set by the Declaration of Helsinki.

### Heart Rate Variability

The participants performed either a “3–3” (3 min in supine position followed by 3 min in standing position) or a “6–6” (6 min supine followed by 6 min standing) orthostatic test immediately after waking up in the morning. The 3–3 test was performed daily, whereas the 6–6 test was performed every third day, using an inter-beat interval (IBI; RR interval) measuring device (watch RS800CX + sensor H7 + chest belt, Polar, Kempele, Finland). The rationale was that the 3–3 procedure diminished the daily constraint and improved comfort for the participants, while the 6–6 procedure, which was more time-consuming, would ensure better reliability (Bourdillon et al., 2017). In the “Results” section, data from both procedures were compiled, as the 3–3 procedure showed satisfying reliability. The participants set up the chest belt and the sensor, set the watch to RR mode, and started recording the RR intervals for 3 (or 6) min in the supine position, immediately followed by 3 (or 6) min in a stationary standing position, in a quiet environment, with relaxed breathing. Recording of the RR intervals ended after the 3 (or 6) min standing.

The RR intervals from the orthostatic tests were first analyzed to remove ectopic beats and or missing heartbeats and other forms of misidentified heartbeats from the recordings. Ectopic beats were then compensated for by means of interpolation to calculate normal-to-normal (NN) intervals. From the NN intervals, the following HRV parameters were extracted: mean HR; the RMSSD; the spectral power in the LF (pLF, 0.04–0.15 Hz) and HF bands (pHF, 0.15–0.40 Hz) in ms^2^; and the values (expressed in normalized units) for LF and HF, labeled nLF and nHF, respectively (Schmitt et al., 2015; Thorpe et al., 2016). The spectral power was estimated using a fast Fourier transform on the resampled NN intervals (4 Hz) using a window length of 250 data points and an overlap of 50%. All computations were performed separately for the supine (SU) and standing (ST) positions using MATLAB^®^ (MathWorks, Natick, MA, United States).

### Baroreflex Sensitivity

The measurements took place in a quiet environment, after 10 min of seated rest, on the last day of BSL and on the first and last days of RCV. Blood pressure was recorded continuously from the index finger for 10 min using a PPG sensor combined with a cuff (Finometer MIDI, Finapres Medical System BV, Enschede, Netherlands). This device was connected to a computer on which the data were recorded at a 1,000-Hz sampling frequency using dedicated software (LabChart Pro, ADInstruments, Oxford, United Kingdom).

Systolic blood pressure (SBP) and IBIs, defined as the intervals between successive systolic peaks, were first extracted from the blood pressure recording. BRS was then calculated using the sequence method, which is based on the identification of at least three consecutive beats in which a strictly defined increase (or decrease) in SBP is followed by a strictly defined increase (or decrease) in the IBI. Fixed minimal changes were considered for SBP and IBI to validate a sequence. Specifically, a minimum change of 1 mmHg between two consecutive SBP values or 5 ms for IBI was set as the smallest increase (or decrease) in a sequence. Furthermore, the minimum correlation coefficient between changes in SBP and IBI to validate a sequence was 0.85 (Parati et al., 1988). Finally, a minimum number of five sequences were set to validate a BRS estimate over the 10 min of recording. For each SBP-IBI trend, the slope of the regression line between changes in SBP and IBI was calculated, and BRS was calculated as the average of all slopes (Bertinieri et al., 1985).

### Accelerometer

During the 3 days of SDP, the participants wore a three-dimensional, low-noise, accelerometer attached to the wrist of the dominant hand (Shimmer3 GSR+, Dublin, Ireland). Data from the accelerometers were checked to ensure that the participants did not fall asleep (even unintentionally) during the SDP period, except during the 3 h authorized daily. Accelerometers were also worn overnight during BSL, SDP, and RCV. These data were used to identify the time windows for PPG signal processing.

### Polysomnography

Sleep was recorded using polysomnography (PSG, Embletta MPR, Ontario, Canada; EMBLA ST+ Proxy, Ontario, Canada) during the last night of baseline, the first and last nights of recovery. PSG included a six-lead electroencephalography (EEG; positioned in F3; C3; O1; F4; C4; O2 according to the international 10–20 system), a two-lead electrooculography (EOG), a three-lead surface electromyography (EMG; right, left, and center chin), a two-lead electrocardiogram (ECG), thoracic (THO), and abdominal (ABD) belts for breathing. Gel was used to improve electrode conductivity (Ten20, Conductive Neurodiagnostic Paste or NuPrep, Skin Prep Gel; Weaver and Company, Aurora, CO, United States). EEG leads were maintained in place using a dedicated head cup. EOG and EMG leads were maintained according to the manufacturer’s instructions (EC2, Electrode Cream-Natus Neurology, Middleton, CA, United States). All PSG recordings were scored by a trained sleep technician using RemLogic 3.4 software (Version 3.4, Embla, Ontario, Canada) and reviewed by certified sleep physicians (Sleep Center Services, Carrollton, TX, United States). Sleep stages were scored according to the 2007 American Academy of Sleep Medicine (AASM) criteria (Iber et al., 2007).

Extracted parameters from PSG were total sleep time (TST in min); sleep efficiency: the percentage of the actual time slept between going to bed and leaving bed in the morning (SE in %); Wake-Up After Sleep Onset (WASO), the duration spent awaken while in bed (WASO in min); Sleep Onset, the duration between going to bed and actual sleep (SO in min); the duration spent in Non-Rapid Eye Movement (NREM) sleep stage 1 (NREM1 in min and in % of sleep time); likewise, for sleep stages 2 and 3 (NREM2 and NREM3, respectively); the duration spent in Rapid Eye Movement sleep stage (REM in min and %); Position Transition: the number of transition from a position of sleep to another (PT, number of occurrence); the Number of Awakening (NA, in number of occurrence).

### Photoplethysmography

PPG recordings were performed overnight using a wearable sensor (Shimmer3 GSR+, Shimmer, Dublin, Ireland) worn by the participants on the first, fourth, and last nights of BSL, each night of SDP, and on the first, third, and last night of RCV. Recordings took place at the participants’ home. The participants were responsible for the installation, start, stop, and removal of the device. Participants were instructed how and when to use the device during their inclusion visit to our laboratory. The PPG probe was placed on the anterior face of the index proximal phalanx of the non-dominant hand; its position was secured using a Velcro wrapped around the phalanx and the probe. The probe was connected with a wire (audio jack) to a small and light box attached to the wrist using a dedicated wristband. Participants returned the box after each night of recording (battery was reloaded and memory emptied in the laboratory). Data were downloaded on a computer using a dedicated software (ConsensysBASIC, Shimmer, Dublin, Ireland), and data quality was immediately checked. Data were then converted to MATLAB format (The MathWorks Inc., Natick, MA, United States) for later analysis. Sampling rate was set at 1,000 Hz. The wearable PPG sensor was light and comfortable to be used overnight without altering sleep quality, as confirmed by the questionnaires (see further).

**FIGURE 2:**
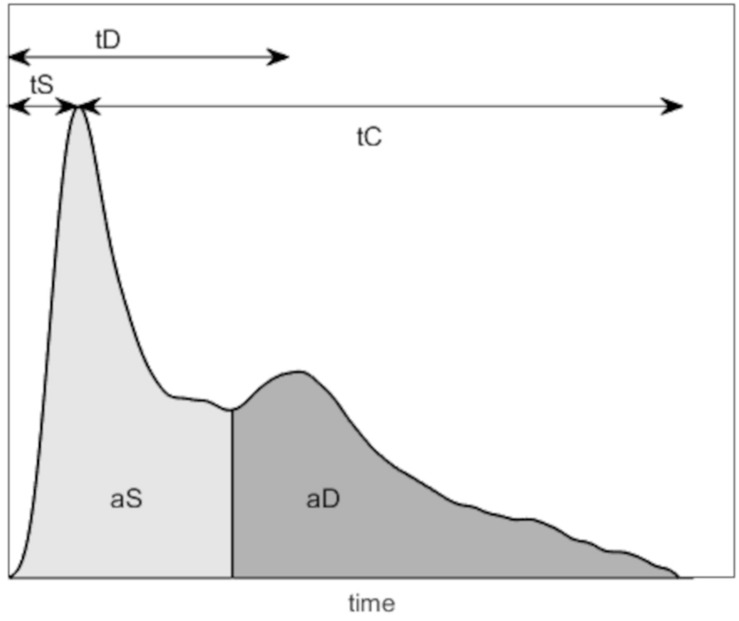
Illustration of the photoplethysmography (PPG) parameters computed. tS, systolic time (s); tD, diastolic time (s); tC, catacrotic time (s); aS, systolic amplitude (mV); aD, diastolic amplitude (mV); aDic, dicrotic notch amplitude (mV).

The PPG waveform was characterized using simple parameters, namely, the mean of pulse interval (PI), aS, aD, systolic time (tS), diastolic time (tD), and catacrotic time (tC). PI, tS, tD, and tC are in seconds; aS and aD are in millivolts. In addition, common PPG parameters were combined to compute a modified augmentation index (mAI) defined in equation 1. For graphic representation of those parameters, refer to Figure 2.

The aS is an indicator of the pulsatile changes in blood volume caused by arterial blood flow at the measurement site (Chua and Heneghan, 2006). It is proportional to the local vascular compliance (Dorlas and Nijboer, 1985) and is related to stroke volume (Murray and Foster, 1996). An increase in aS is associated with peripheral vasodilation and increased stroke volume while a decrease is associated with increased peripheral resistance and vasoconstriction (Korhonen and Yli-Hankala, 2009).

The aD is informative about the relaxation phase of the cardiac cycle and the vascular tone. An increase is associated with relaxed arteries, while a decrease is associated with increases in arterial stiffness or vascular tone (Millasseau et al., 2002; Elgendi, 2012).

The mAI is associated with reduced compliance of the elastic arteries, which may result in increased left ventricular after load, decreased diastolic blood pressure, and impaired perfusion (Takazawa et al., 1998). mAI is defined as:

$$m\,A\,I\,\frac{1}{n}\,\sum_{1}^{n}\frac{a\,D-a\,D\,i\,c}{a\,S-a\,D\,i\,c}$$

where aD and aS are as defined previously, and aDic is the amplitude of the dicrotic notch, as shown on Figure 2.

The PPG values reported were averaged over the last hour of sleep of the recorded night. The rationale for this method is based on the differences of nights’ duration between the three phases, i.e., normal (during BSL), shorter (during SDP), or longer (during RCV) than usual. Therefore, comparison of full overnight recordings or with respect to sleep stages was meaningless given that the amount of data and structure of sleep stages were drastically different between conditions. The last hour of sleep was detected by the data from the accelerometer worn at the wrist (Shimmer3 GSR+, Shimmer, Dublin, Ireland; read further). This particular window was determined using common algorithms, starting from sleep onset and ending with waking up after the last cycle of sleep (van Hees et al., 2018). These algorithms are based on the magnitude of accelerations (Borazio et al., 2014) and changes of arm angle (van Hees et al., 2015).

### Psychomotor Vigilance Task

The participants performed a psychomotor vigilance task (PVT-192, CWE Inc., PA, United States) for 10 min on the last day of BSL, each day of SDP, and days 1, 3, and 7 of RCV; the task consisted of pressing a designated button as soon as a timer appeared on the device screen. When the button was pushed, the timer stopped, and the participants could see their response time in milliseconds. The timer started randomly to prevent anticipation. The mean and SD of response times (RT and SD-RT, respectively) were used to identify changes in vigilance.

### Questionnaires

Each day, the participants answered the eight-item overtraining (Atlaoui et al., 2004), the Karolinska Sleepiness Scale (KSS; Akerstedt and Gillberg, 1990), and the Groningen Sleep Quality Scale (GSQS; Jafarian et al., 2008) questionnaires. Those questionnaires were filled in the morning, ideally after breakfast and before any other activity in the day. The score of the eight-item corresponded to the sum of the participants’ responses to each item. The score of the KSS corresponds to the participants’ rating. The score of the GSQS was obtained according to Jafarian et al. (2008).

On days 1, 4, and 7 of BSL, every day during SDP, and on days 1, 4, and 7 of RCV, the participants were required to fill the 14-item fatigue scale (Chalder et al., 1993) and Profile of Mood States (POMS) questionnaires, in addition to the other questionnaires. The 14-item questionnaire contains eight items corresponding to physical signs and six corresponding to mental signs of fatigue. The score is divided between physical and mental signs of fatigue. The POMS questionnaire provides scores for anger, confusion, depression, fatigue, tension, and vigor. For all questionnaires, the lower the score, the greater the participant’s self-perceived well-being; the higher the score, the higher the perception of fatigue, sleepiness (or mood states, except vigor for the POMS).

### Statistics

Parameters recorded every day or every third day were analyzed across days in the following manner: BSL values were the average of the 7 days of baseline. SDP values were recorded daily but not averaged (SDP1, SDP2, and SDP3). RCV values were recorded separately for days 1 and 2 of RCV, and the subsequent values (from day 3 to 7 of RCV) were averaged (RCV1, RCV2, and RCV3–7, respectively). Such method allows the presentation of the acute changes (i.e., during SDP and during the first days of RCV) of the different responses and therefore a clearer display of their time course in the SPD and RCV phases.

All results are given as mean ± standard deviation (SD). For better clarity, standard error of mean (SEM) is plotted on all figures. The Shapiro–Wilk test was used to ensure normality of the data. Measurements were evaluated with a linear mixed-effects analysis for time effect. Tested time points were BSL, SDP and RCV or BSL, SDP1, SDP2, SDP3, RCV1, RCV2, and RCV3–7 depending on the collected parameter. Fixed effects included time, while participant was set as a random effect. Statistical analyses were performed using MATLAB (R2019a, The MathWorks Inc., Natick, MA, United States). The statistical power of the performed tests was set at alpha = 0.05 for significance and alpha = 0.10 for tendency. For tendencies, actual *p*-values are reported, while for significances, actual *p*-values are reported. The Tukey–Kramer *post-hoc* was used when appropriate. Effect sizes are reported using Cohen’s *d* = (x́_1_−x́_2_)/s, with x́_1_∧ x́_2_ the means of the two populations being compared and s the combined standard deviation of the two populations. Effect size was considered small when d < 0.20, medium when 0.20 ≤ d < 0.80, and large when d ≥ 0.80.

HRV and PPG were set up by the participants at home. Participants were instructed on the best practice for recordings and had feedback on the signal quality to ensure the best recording possible. Inter-operator variability was negligible. All the other setups were made by a single operator to avoid any inter-operator variability.

## Results

Fifteen participants (eight women, seven men) completed the entire study (age 22 ± 2 years, height 173 ± 9 cm, weight 66 ± 12 kg).

Results for HRV are shown in Table 1 for the supine position and in Table 2 for the standing position. Mean HR did not change throughout the protocol (main effect *p* > 0.52 and *p* = 0.58 for supine and standing positions, respectively). HF decreased in the standing position in SDP1, SDP2, and SDP3 compared to BSL (main effect *p* = 0.04, BSL vs. SDP1, SDP2, and SDP3, *d* = 0.25, *d* = 0.26, and *d* = 0.24, respectively).

**TABLE 1:** Heart rate variability (HRV) parameters in supine position.

	Supine						
	
	BSL	SDP1	SDP2	SDP3	RCV1	RCV2	RCV3–7
HR (bpm)	616	607	626	615	649	639	635
RMSSD (ms)	6812	7219	7023	6822	6916	6521	6519
LF (ms^2^)	4,8831,988	5,0683,836	5,5176,292	3,8512,472	3,8623,029	4,2123,486	4,2442,713
HF (ms^2^)	3,9852,028	5,1826,382	4,1034,180	3,0233,335	3,2693,245	4,4694,636	4,3913,665
Total power (ms^2^)	12,3264,933	14,48812,231	14,88217,635	10,2635,810	10,9108,620	11,2169,006	11,8307,752
nLF	0.570.07	0.570.19	0.600.18	0.620.17	0.570.20	0.520.14	0.540.11
nHF	0.430.07	0.430.19	0.400.18	0.380.17	0.430.20	0.480.14	0.460.11

**Mean ± SD. HR, heart rate; RMSSD, root mean square of the standard deviation of the inter-beat intervals; LF, power in the low-frequency band; HF, power in the high-frequency band; nLF, normalized power of the low-frequency band; nHF, normalized power of the high-frequency band.**

**TABLE 2:** Heart rate variability (HRV) parameters in standing position.

	Standing						
	
	BSL	SDP1	SDP2	SDP3	RCV1	RCV2	RCV3–7
HR (bpm)	918	929	958	938	918	9412	926
RMSSD (ms)	3316	2710*	2514*	278*	2914	3430	3112
LF (ms^2^)	2,8701,519	3,0091,752	2,0661,578	2,375850	2,3181,058	3,4514,603	2,7121,130
HF (ms^2^)	646618	358*209	366*382	386*198	445384	9001,856	548538
Total power (ms^2^)	5,8803,340	5,6882,962	3,9083,260	4,6261,717	4,3181,804	7,8621,846	5,3552,814
nLF	0.850.04	0.890.04*	0.860.05	0.860.04	0.850.08^*a*^	0.860.06^*a*^	0.850.04^*a*^
nHF	0.150.04	0.110.04*	0.140.05	0.140.04	0.150.08^*a*^	0.140.06^*a*^	0.150.04^*a*^

Mean ± SD. HR, heart rate; RMSSD, root mean square of the standard deviation of the inter-beat intervals; LF, power in the low-frequency band; HF, power in the high-frequency band; nLF, normalized power of the low-frequency band; nHF, normalized power of the high-frequency band. *Different from BSL; ^*a*^Different from SDP1 (p < 0.05).

BRS and BP did not change throughout the protocol. BRS main effect *p* = 0.87: BSL: 13.9 ± 4.8, RCV1: 13.8 ± 5.0, RCV7: 14.7 ± 6.8 ms/mmHg. BP systolic main effect *p* > 0.53: BSL: 114 ± 22, SDP: 110 ± 16, RCV: 107 ± 23 mmHg. BP diastolic main effect *p* > 0.64: BSL: 61 ± 13, SDP: 57 ± 13, RCV: 59 ± 18 mmHg.

**FIGURE 3:**
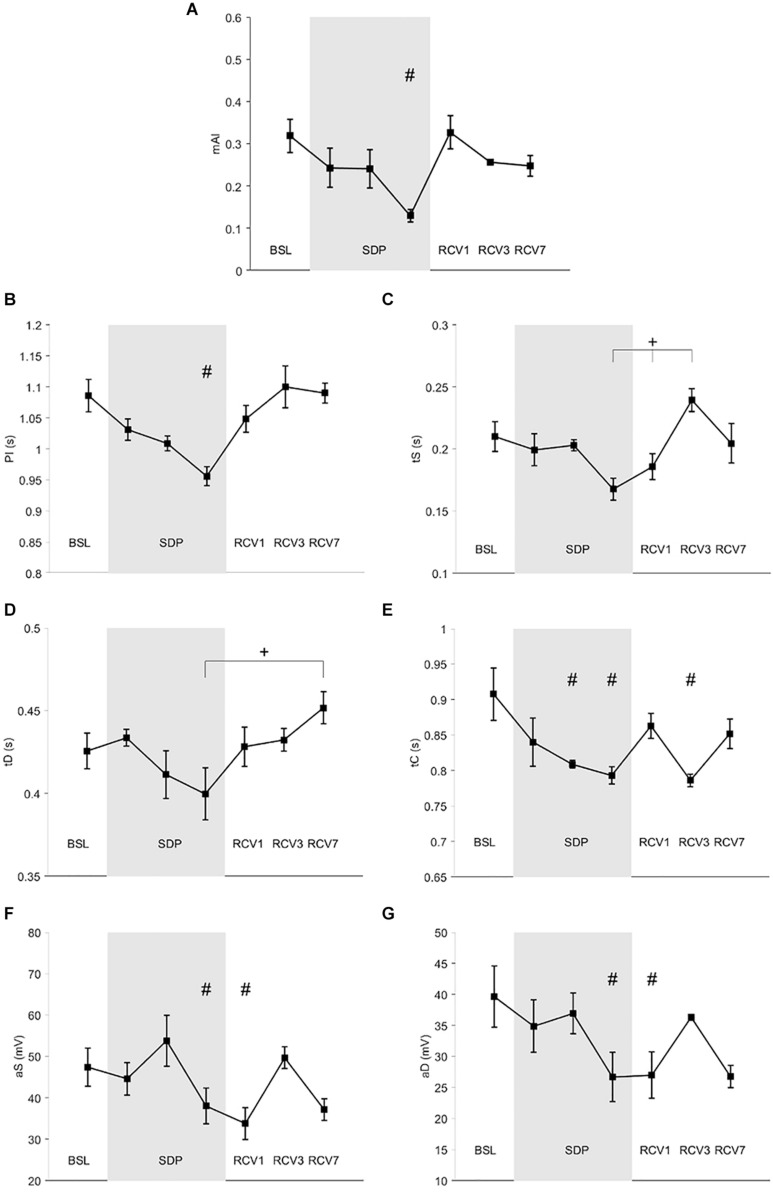
Photoplethysmography (PPG) parameters during baseline (BSL), sleep deprivation (SDP), and recovery (RCV) periods. **(A)** mAI, modified augmentation index; **(B)** PI, pulse interval; **(C)** tS, time of systolic wave; **(D)** tD, time of dicrotic wave; **(E)** tC, time of catacrotic wave; **(F)** aS, systolic amplitude; **(G)** aD, diastolic amplitude. # different from BSL (*p* < 0.05), + difference between time points (*p* < 0.05).

Results for PPG are shown in Figure 3. mAI and PI (panels A and B, respectively) decreased in SDP3 compared to BSL (main effect *p* = 0.003, *d* = 2.05 and *p* = 5.10^–5^, *d* = 1.93, respectively). tS (panel C) increased from SDP3 and RCV1 to RCV3 (main effect *p* < 0.002, *d* = 0.69 for SDP2 vs. RCV1 and 0.60 for SDP3 vs. RCV1). tD (panel D) increased from SDP3 to RCV7 (main effect *p* = 0.048, *d* = 1.27). Catacrotic time (panel E) decreased in SDP2, SDP3, and RCV3 compared to BSL (main effect *p* = 0.003, *d* = 1.19 for SDP2 vs. BSL, *d* = 1.32 for SDP3 vs. BSL, and *d* = 1.44 for RCV3 vs. BSL). aS and aD (panels F and G, respectively) decreased in SDP3 and RCV1 compared to BSL (main effects systolic *p* = 0.01; *d* = 0.66 for SDP3 vs. BSL and *d* = 1.01 for RCV1 vs. BSL; diastolic *p* = 0.02, *d* = 0.91 for SDP3 vs. BSL and *d* = 0.91 for RCV1 vs. BSL).

Results for PSG are shown in Table 3. Total sleep time (TST) and sleep efficiency (SE) increased in RCV1 (i.e., the first long night after sleep deprivation) compared to BSL, while wake-up after sleep onset (WASO) decreased (TST: *p* = 6.10^–4^, *d* = 1.37; SE: *p* = 0.001, *d* = 1.32; WASO: *p* < 0.001, *d* = 1.07, respectively). Duration of REM sleep in absolute and relative value increased in RCV1 compared to BSL (*p* = 0.002, *d* = 1.24 and *p* = 0.01, *d* = 0.78, respectively), while the number of awakening decreased (*p* = 0.03, *d* = 0.72).

**TABLE 3:** Polysomnography parameters.

	BSL	RCV1	RCV7
TST (min)	379.547.9	427.958.3*	401.443.9
SE (%)	79.710.2	89.212.1*	83.69.2
WASO (min)	58.449.7	7.17.8*	44.731.4
SO (min)	37.626.8	44.957.4	33.837.5
NREM1 (min)	8.44.3	6.45.2	7.93.1
NREM1 (%)	2.21.2	1.71.9	2.00.8
NREM2 (min)	236.941.5	245.139.6	236.434.8
NREM2 (%)	62.57.6	57.46.5	59.07.2
NREM3 (min)	56.220.4	72.025.9	61.122.8
NREM3 (%)	14.95.0	16.75.5	15.35.7
REM (min)	78.121.0	104.426.4*	97.526.2*
REM (%)	20.34.8	24.24.4*	23.94.4*
PT (n)	23.714.3	16.312.2(*)	17.97.4
NA (n)	6.53.1	3.92.7*	6.33.2

Mean ± SD. TST, total sleep time; SE, sleep efficiency; WASO, wake up after sleep onset; SO, sleep onset; NREM 1, 2, and 3, non-REM sleep stages 1, 2, and 3; REM, rapid eye movement; PT, position transition; NA, number of awakening. *Different from BSL (p < 0.05).

**FIGURE 4:**
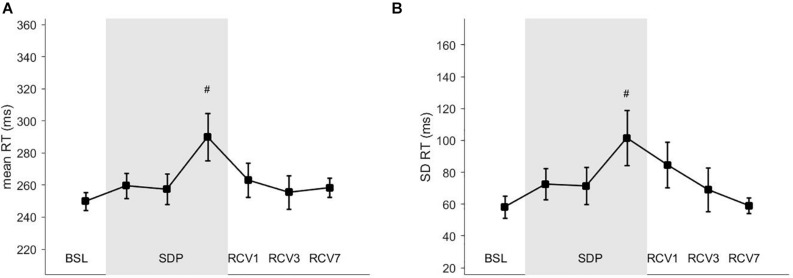
Vigilance task parameters. **(A)** RT, mean reaction time; **(B)** SD RT, standard deviation of the reaction time. # different from BSL (*p* < 0.05).

Results for PVT are shown in Figure 4, mean reaction time (panel A) increased at SDP3 compared to BSL (*p* = 0.02, *d* = 0.02). Standard deviation of reaction time (panel B) also increased at SDP3 compared to BSL (*p* = 0.04, *d* = 0.01).

**FIGURE 5:**
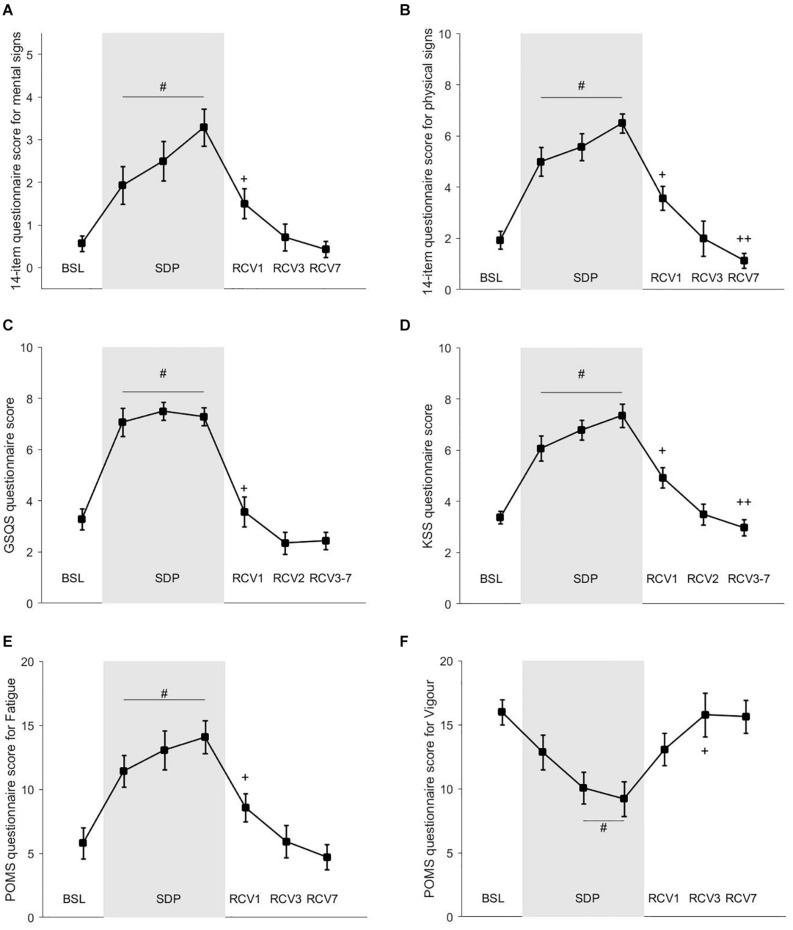
Questionnaires’ scores. **(A)** 14-item questionnaire score for mental sign of fatigue; **(B)** 14-item questionnaire score for physical sign of fatigue; **(C)** GSQS questionnaire score; **(D)** KSS questionnaire score; **(E)** POMS questionnaire score for Fatigue; **(F)** POMS questionnaire score for vigor. # different from baseline (BSL; *p* < 0.05). + different from sleep deprivation (SDP)3 (*p* < 0.05). ++ different from recovery (RCV)1 (*p* < 0.05).

Scores from questionnaires are shown in Figure 5. Sleepiness according to KSS (panel D) increased in SDP1, SDP2, and SDP3 compared to BSL (main effect *p* = 10^–16^, BSL vs. SDP1, SDP2, and SDP3: *d* = 1.02, *d* = 1.95, and *d* = 1.75, respectively). Quality of sleep according to GSQS (panel C) decreased (GSQS score increased) in SDP1, SDP2, and SDP3 compared to BSL (main effect *p* = 10^–20^, BSL vs. SDP1, SDP2, and SDP3: *d* = 1.05, *d* = 1.92, *d* = 1.84, respectively). Fatigue according to the eight items increased in SDP1, SDP2, and SDP3 compared to BSL (main effect *p* = 10^–20^, BSL vs. SDP1, SDP2, and SDP3: *d* = 0.13, *d* = 0.25, and *d* = 0.26, respectively). Physical signs of fatigue according to the 14 items (panel B) increased in SDP1, SDP2, and SDP3 compared to BSL (main effect *p* = 9.10^–16^, BSL vs. SDP1, SDP2, and SDP3: *d* = 0.88, *d* = 1.11, and *d* = 2.30, respectively). Mental signs of fatigue according to the 14 items (panel A) increased in SDP1, SDP2, and SDP3 compared to BSL (main effect *p* = 4.10^–9^, BSL vs. SDP1, SDP2, and SDP3: *d* = 0.65, *d* = 0.83, *d* = 1.36, respectively). Fatigue according to POMS (panel E) increased in SDP1, SDP2, and SDP3 compared to BSL (main effect *p* = 2.10^–12^, BSL vs. SDP1, SDP2, and SDP3: *d* = 0.25, *d* = 0.25, and *d* = 0.35, respectively). Vigor according to POMS (panel F) decreased in SDP1, SDP2, and SDP3 compared to BSL (main effect *p* = 10^–8^, BSL vs. SDP1, SDP2, and SDP3: *d* = 0.14, *d* = 0.31, and *d* = 0.31, respectively).

## Discussion

This study investigated the changes in HRV, PPG, and BRS during partial sleep deprivation. The main results are that both HRV and PPG are modified during SDP, contradictory to BRS: during the 3 days of sleep deprivation when compared to baseline, there were changes in HRV (decrease in HF power associated with an increase in nLF) and in PPG (decreases in amplitude and duration of the PPG waveform), which indicate decreased parasympathetic and maybe increased sympathetic modulations, while BRS remained unchanged. During recovery, HRV and PPG showed an acute restoration of the parasympathetic activation.

### Sleep Deprivation as a Stressor

Sleep deprivation is usually seen as a stressor (McEwen and Karatsoreos, 2015) and is expected to increase the sympathetic activity (Zhong et al., 2005) and the hypothalamic–pituitary–adrenal axis (Wright et al., 2015). However, previous studies investigating HRV under sleep deprivation reported discrepant results. Sleep timing, circadian rhythmicity, posture, physical activity, and other factors influence HRV parameters, which make their interpretation difficult. In the present study, we standardized the HRV measures with an orthostatic test. The HRV parameters reported are compatible with a shift toward increased sympathetic and decreased parasympathetic modulations during sleep deprivation, which seems to recover acutely during recovery.

### Heart Rate Variability and Photoplethysmography Indicate Parasympathetic Withdrawal

The changes in HRV indicate that sleep deprivation was intense enough to inhibit vagal modulations to the heart (decreased HF). This result was in accordance with previous literature (Tobaldini et al., 2013; Morales et al., 2019) and has been associated with increased blood biomarkers, such as adrenaline, norepinephrine (Dettoni et al., 2012), dopamine, thyroid-stimulating hormone (Chapotot et al., 2001; Wright et al., 2015), and cortisol (Schwarz et al., 2018). However, in the present study, the decrease in parasympathetic modulations was clear while the increase in sympathetic modulations was less evident, which indicates a parasympathetic withdrawal rather than a sympathetic over-activation during partial sleep deprivation.

The PPG results were compatible with HRV, indicating a withdrawal of parasympathetic modulations. Specifically, the decrease in mAI could be associated with decreased parasympathetic modulations or increased sympathetic modulation. This is consistent with the decrease in PI in SDP3 (PI, Figure 3), which correlates with an increase in heart rate during sleep and therefore could be associated with the parasympathetic withdrawal. In addition, the catacrotic time (tC, Figure 3), which corresponds to the relaxation phase of the heart cycle, also decreased (in SDP2 and SDP3), which may be linked to either a parasympathetic withdrawal or an increased sympathetic drive. During SDP3 and RCV1, there was a decrease in the aS and aD, which is associated with increased vessel tone, vasoconstriction (Korhonen and Yli-Hankala, 2009), and peripheral resistance (Elgendi, 2012). Again, this observation is compatible with a parasympathetic withdrawal or a sympathetic over-activation.

During the recovery phase, there was an increase in tS and tD and therefore in PI (Figure 3), which is compatible with a return of the parasympathetic modulations on the heart, slowing down the heart rate. Decreased vessel tone and lengthening of the PPG wave may rather be due to a decreased sympathetic drive (Millasseau et al., 2002).

BRS did not change throughout the protocol, which, to our knowledge, is a new finding. This is consistent with previous studies reporting no change in blood pressure after partial sleep deprivation, although BRS was not reported in those studies (Arnal et al., 2015). In the present study, resting BP did not change significantly after partial sleep deprivation, suggesting that it was adequately buffered by the baroreflex arc (Dettoni et al., 2012) despite the present acute partial sleep deprivation. Generally, sleep deprivation elevates blood pressure and may alter BRS when it is chronic (i.e., persistent partial sleep deprivation for months as a lifestyle or in night shift workers) (Gangwisch et al., 2006) or during the night (Yang et al., 2019).

### Complementarity of Heart Rate Variability and Photoplethysmography

HRV holds information on the autonomous regulation of the heart, while PPG holds information on the peripheral circulation. Although HRV and PPG are intimately linked, they are informative on different aspects of the autonomic balance and cardiovascular functions. HRV is mostly under central influences while PPG is under both central (autonomic balance) and peripheral (local control of vascular tone) influences; therefore, the mechanisms of regulation are distinct. PPG and HRV are complementary means of monitoring (Bourdillon et al., 2019), and in the present work, they both indicate a parasympathetic withdrawal. It is of practical interest for the clinician to now have the possibility to record accurately and non-invasively both PPG and HRV with low-cost wearables.

### Sleep Deprivation Impaired Cognitive Function and Fatigue Sensation

There was a rebound of sleep duration, efficiency, and REM sleep in RCV1 compared to SDP, which was expected (Berry and Wagner, 2015) and confirms the state of sleep deprivation of the participants. The state of fatigue of the participants was also confirmed by the questionnaires, which showed an increased sensation of physical and mental fatigue (8 and 14 items), sleepiness (GSQS and KSS), and a decrease in vigor (POMS). This is consistent with previous studies that demonstrated a negative correlation between the parasympathetic markers of HRV and depression, anxiety, and hostility. Similarly, there is a positive correlation of the sympathetic markers of HRV and the same parameters (Morales et al., 2019).

Additionally, in line with the literature for confirming the effects of sleep deprivation, both reaction time and consistency of the responses to stimuli increased during the vigilance performance task, which indicate impaired cognitive functions (Nir et al., 2017). Occupational medicine should focus on sleep deprivation prevention to reduce the risks associated, such as hypertension (Gangwisch et al., 2006), infarctus and stroke (Ayas et al., 2003), and attention deficit (Arnal et al., 2015).

### Limitations

To our knowledge, the present article is the first one to combine the use of several complementary non-invasive cardiovascular markers to assess the effects of partial sleep deprivation. However, it has some limitations: BRS was measured during RCV1 and not SDP3, which may have mitigated the effects of sleep deprivation.

In addition, the reported parameters of the PPG waveform may be related to each other, as for example reductions in tS and tD are likely to arise from reduction in PI. However, the complex nature of the PPG waveform may alter these relationships; hence, it is relevant to present parameters separately, showing that one parameter can be significantly altered without changes in the others. PPG is a low-cost and non-invasive technique coupled with simple signal processing. Although light and comfortable, the experimental device used could be designed to be more ergonomic and user-friendly in the future, allowing reliable overnight PPG recordings at minimal cost and inconvenience.

Although the timing and duration of sleep deprivation were closely controlled in the present study, we cannot exclude that going to sleep late and waking up early did not shift the circadian rhythm of the participants. This may be a confounding factor to sleep deprivation *per se*; however, the two factors are likely closely linked and dependent on one another. Practically, measuring the effects of both concomitantly is relevant.

In this study, the sample size of 15 participants is rather small compared to the effect size reported on HRV. Future studies shall include more participants for better effect size.

The menstrual phase of the female participants was not controlled in this study. This may have influenced the results, but assessing the effects of the menstrual cycle was beyond the scope of this study.

The participants breathed spontaneously during the HRV and BRS recordings. Imposing a breathing pattern would probably have decreased the inter- and intra-participant variability, but it would have also altered the natural breathing pattern and its influence on both HRV and BRS, therefore potentially masking a natural modification due to sleep deprivation. One limitation of the present study was that we did not record the respiratory rate, which may have influenced the frequency bands.

## Conclusion

HRV- and PPG-extracted parameters are markers easily measured with wearable devices and modified by partial sleep deprivation [i.e., 3 h of sleep per night (SDP) during three consecutive nights]. Those markers showed a decrease in parasympathetic activity, known as detrimental to the cardiovascular health. Moreover, BRS remained unchanged while vigilance decreased during SDP. Overall, the present study reports that all these measurements have their own time course and are complementary. The clinical usefulness of our findings requires further investigation.

## Data Availability Statement

The datasets generated for this study can be found in the online repositories. The names of the repository/repositories and accession number(s) can be found below: https://zenodo.org/record/4326598#.X9nH0NhKiUk.

## Ethics Statement

The studies involving human participants were reviewed and approved by the Agreement 2016-00308; Commission Cantonale d’Ethique de la Recherche sur l’être humain, CCER-VD; Lausanne, Switzerland. The patients/participants provided their written informed consent to participate in this study.

## Author Contributions

NB and GM designed the study. NB, PA, and PH conducted the experiments. NB analyzed the data, wrote the article, and prepared the figures. MN and FJ did the signal processing and data analysis for PPG. GM reviewed the article. All the authors approved the final version of the manuscript.

## Conflict of Interest

MN and FJ are employees of my-vitality sàrl. NB is an employee of be.care SA. The remaining authors declare that the research was conducted in the absence of any commercial or financial relationships that could be construed as a potential conflict of interest.
